# Hyperthyroidism with concurrent FMS-like tyrosine kinase 3-internal tandem duplication-positive acute promyelocytic leukemia: A case report and review of the literature

**DOI:** 10.3892/ol.2013.1721

**Published:** 2013-11-29

**Authors:** YAJIAN JIANG, KEYUE HU, WANZHUO XIE, GAOFENG ZHENG, JIE SUN, YANLONG ZHENG, HE HUANG

**Affiliations:** 1 Department of Hematology, Bone Marrow Transplant Center, The First Affiliated Hospital of Zhejiang University Medical School, Hangzhou, Zhejiang 310003, P.R. China; 2 Program in Clinical Medicine, School of Medicine, Zhejiang University, Hangzhou, Zhejiang 310029, P.R. China; 3 Department of Hematology, The Second People’s Hospital, Ningbo, Zhejiang 315000, P.R. China

**Keywords:** hyperthyroidism, anti-thyroid drugs, acute promyelocytic leukemia, FMS-like tyrosine kinase 3-internal tandem duplication

## Abstract

Neutropenia is a common side-effect in hyperthyroid patients with long-term use of antithyroid drugs. This may be caused by drug-induced immune dysfunction or increased thyroxine hematologic toxicity, which usually returns to normal after medication is discontinued or the hyperthyroidism becomes well controlled. However, hyperthyroidism with pancytopenia is extremely rare. The current case report presents a hyperthyroid patient complicated with pancytopenia who had taken antithyroid drugs for 14 years. Bone marrow analysis revealed primary leuokocytes, indicating M3 acute leukemia. Genetic analysis revealed promyelocytic leukemia-retinoic acid receptor α fusion and FMS-like tyrosine kinase 3-internal tandem duplication. The genetic abnormality was also associated with thyroid hormonal functions. After a standard anti-M3 regimen was administed, the patient achieved complete remission and maintained stable thyroid functions. To the best of our knowledge, this is the first reported case of a patient with hyperthyroidism acquiring M3 leukemia harboring the FMS-like tyrosine kinase 3-internal tandem duplication.

## Introduction

Hyperthyroidism is a condition in which the thyroid gland releases excessive thyroid hormones into the blood circulation. A number of diseases, including Graves’ disease and thyroid adenoma, can lead to the hyperthyroidism state (1). Among patients with hyperthyroidism, 5–20% present with a low leukocyte count in peripheral blood. Although the exact mechanism has not been elucidated, autoimmune reaction, high metabolism, thyroid hormonal effects and other factors have been proposed to make major contributions to the decreased leukocyte count in these patients (2). Furthermore, antithyroid treatment received by these patients may make them prone to neutropenia, a condition which can further deteriorate to granulocytopenia or agranulocytosis. However, these conditions usually resolve when medication is discontinued or the hyperthyroidism is brought under control (3).

Single hematological lineage abnormalities have been reported in hyperthyroidism, however, pancytopenia is a rare presentation of hyperthyroidism. Hyperthyroidism complicated with leukemia is even rarer, particularly acute promyelocytic leukemia (APL) with FMS-like tyrosine kinase 3-internal tandem duplication (FLT3-ITD). The c-erb-A protooncogene, a thyroid hormone receptor analog, is located in the specific chromosomal translocation t(15;17) in APL. The oncogene can suppress the activity of a responsive promoter. However, when thyroid hormone is added in *in vitro* systems, expression of the promotor is stimulated (4).

The following case report presents a male patient with hyperthyroidism who had taken antithyroid drugs for 14 years prior to the onset of pancytopenia. The bone marrow assay was consistent with French-American-British (FAB) classification criteria for APL diagnosis, later confirmed by the presence of promyelocytic leukemia-retinoic acid receptor α (PML-RARα) fusion gene, concurrent with FLT3-ITD mutation. All-trans retinoic acid (ATRA) treatment was effective in this patient in correcting the pancytopenic state. To the best of our knowledge, this is the first case report of hyperthyroidism concurrent with FLT3-ITD-positive APL, and we hypothesize that the translocated genetic abnormity involving the c-erb-A protooncogene may play a major role in the leukemogenesis. Written informed consent was obtained from the patient.

## Case report

A 42-year-old male patient was admitted to The First Affiliated Hospital of Zhejiang University School of Medicine (Hangzhou, China) in September 2012, complaining of fever, sore gums and repeated episodes of fatigue. The patient had a history of hyperthyroidism for 14 years and had been treated with propylthiouracil (PTU) since diagnosis. Due to drug-related leukopenia, the patient’s medication was changed from PTU to methimazole (MMI) in 2010. Four months prior to admission, no abnormal thyroid hormone levels or decreased peripheral blood counts were revealed.

**Figure 1:**
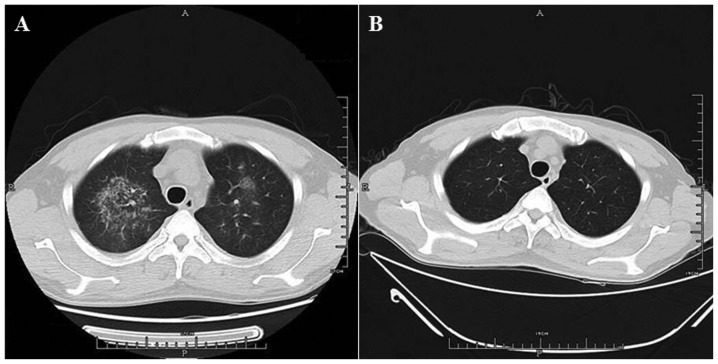
Chest computer tomography scan. (A) Interstitial changes and small quantity of pleural effusions in the lungs prior to treatment. (B) Improvement in the lungs following effective treatment in the patient.

Upon admission, the patient had a pale anemic appearance and mildly bulging eyeballs. Vital signs were stable. Hepatomegaly, splenomegaly and enlarged surface lymph nodes were not identified. Pancytopenia was revealed by the following pathology results: Leukocyte count, 1,600 cells/μl, with 6.25% neutrophils; hemoglobin levels (Hb), 8.6 g/dl; and platelet (PLT) count, 51,000 PLT/μl. Thyroid hormone levels were as follows: Total triiodothyronine levels, 0.69 nmol/l; free triiodothyronine, 2.25 pmol/l; and thyroid peroxidase antibody, 357.70 IU/ml. Thyroid-stimulating hormone levels were reported as normal. As the patient had a mild cough, a computer tomography (CT) scan was performed revealing interstitial changes in the lungs with a small quantity of pleural effusions (Fig. 1).

**Figure 2:**
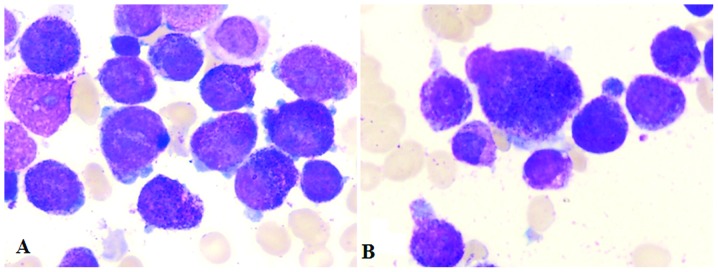
Wright-Giemsa staining of the aspirated bone marrow smear indicating a hypercellular state and significant active myeloid hyperplasia. Abnormal promyelocytic blast cells with increased cytoplasm particles are shown.

In agreement with endocrinologists, administration of MMI was terminated and granulocyte colony-stimulating factor (G-CSF) and empirical antibiotics were prescribed. However, the patient refused a bone marrow aspiration assay until three days following the use of G-CSF. Assay results demonstrated a hypercellular state, with 16.5% large blast cells and 54.5% abnormal promyelocytic cells, consistent with diagnosis of acute myeloid leukemia (AML) M3a using FAB classification (Fig. 2). Genetic tests reported PML-RARα, t(15;17) translocation and FLT3-ITD mutation. Immediately, the patient was treated with ATRA (25 mg/m^2^/d, *per os*). Twenty-one days following ATRA treatment, the blood cell count showed signs of recovery: Leukocyte count, 1,500 cells/μl; Hb, 9.6 g/dl; PLT count, 44,000 PLT/μl. Chest symptoms were also improved and later confirmed by chest CT (Fig. 1). Twenty-eight days following ATRA treatment, blood cell count was normal and a bone marrow smear indicated remission. Thyroid function remained normal while the antithyroid therapy was discontinued. Therefore, the patient’s endocrinologist advised that thyroid conditions be monitored without other management. The patient was enrolled into consolidation chemotherapy for APL and remains in remission to date.

## Discussion

Hyperthyroidism is a condition which involves elevated thyroid hormones in the blood circulation. A number of diseases, including Graves’ disease, thyroid adenoma and thyroiditis, may lead to hyperthyroidism (1,5). PTU and MMI are two major antithyroid drugs used in hyperthyroidism patients which function through inhibiting the synthesis of thyroid hormones. Furthermore, PTU can prevent the conversion of T4 to T3. When antithyroid drugs fail, or under specific conditions, other treatment choices include radioactive iodine and thyroid surgeries (6).

Thyroid diseases, particularly those caused by autoimmune reactions, have long been observed to be associated with hematological diseases, including lymphoid hyperplasia, chronic lymphocytic leukemia (CLL) and non-Hodgkin’s lymphoma (7–10). The first case reporting a correlation between hyperthyroidism and leukemia dates back to 1924, when CLL and Graves’ disease were reported in a single patient and thyroid removal resulted in complete remission of the two diseases (11). A retrospective study of patients between 1978 and 1989 revealed that 3.1% (27/870) of patients with acute leukemia had evidence of thyroid disease. Twenty-one patients had AML, five had acute lymphoid leukemia, and one had accelerated chronic myeloid leukemia (12). Thyroid diseases, particularly autoimmune thyroid diseases, can accompany a dysregulated immune system which may produce disordered immune reactions which lead to damage of the bone marrow (13). Another possible link between thyroid disease and leukemia concerns growth regulation at the molecular level. The thyroid hormone receptor and the retinoid receptor belong to the same family, and the DNA binding domains of the two receptors have a 62% identical amino acid sequence (14). Furthermore, the oncogene analog of the thyroid hormone receptor, c-erb, may function in the regulation of cell growth (4). Although a number of possible causes have been proposed, the exact mechanism is not clear.

The long-term use of antithyroid drugs in patients with hyperthyroidism can usually complicate the hematological conditions caused by the disease itself or induced by the drugs. The major adverse effects of the two drugs broadly used in hyperthyroidism, PTU and MMI, are rash, urticaria, joint pain, stomach intolerance, agranulocytosis disease and liver toxicity (15). Furthermore, PTU can lead to production of cytoplasmic antibodies and vasculitis (12,16). With regard to hematological disorders, PTU is known to cause bone marrow aplasia and AML (17,18). Although the underlying mechanism remains unclear, it has been proposed to involve an immune reaction to the bone marrow stem cells, thus causing hematological disorders. In addition, a direct toxic effect to the bone marrow stem cells may be responsible for the induced hematological disorders (19).

The present patient had hyperthyroidism and was prescribed PTU for 11 years. It is unlikely that the hematological malignancy in this report was caused by long-term treatment with PTU. However, the patient’s medication was changed to MMI three years prior to the onset of the aforementioned symptoms. This is not consistent with the report that side effects, including agranulocytosis, pancytopenia and certain other hematological disorders, often occur within the first three months following intake of an antithyroid drug (13).

The patient developed APL, which has been suggested to be the most curable type of AML, following the administration of MMI for hyperthyroidism (20). APL is often primary, but can be secondary to other diseases, including myelodysplastic syndrome. APL is characterized by abnormal promyelocytic hyperplasia with numerous Auer rods, t(15;17) translocation leading to PML-RARα rearrangement, and specific differentiation of blast cells by ATRA (20). Notably, the aforementioned c-erb-A protooncogene is located in the region of the t(15;17) translocation of APL. This hormone receptor can stimulate growth when thyroid hormone is added *in vitro*(4).

In addition, in the current patient, the FLT3-ITD mutations were confirmed by genetic testing. The FLT3 gene is a growth factor receptor gene with tyrosine kinase activity. It is closely associated with the occurrence of leukemia. It is currently speculated that the FLT3 gene, co-expressed with its receptor in leukemic cells, can promote the proliferation and maintenance of the leukemic clone by autocrine and paracrine pathways. FLT3-ITD mutations can be used as important prognostic indicators of AML. FLT3-ITD-positive AML is often associated with poor prognosis (21), often resulting in the recurrence of AML (22).

The current patient developed APL during the course of hyperthyroidism. Association of the two diseases should be suspected in the first instance based on the correlations between thyroid disease, antithyroid drugs and hematological disorders. The onset of symptoms were noted three months following the change from PTU to MMI. The adjustment from PTU to MMI may contribute to fluctuations of thyroid hormones, which resulted in APL. Another possibility of APL in this patient may be due to the toxicity of MMI. However, the exact reason cannot be elucidated. For this patient with APL, the FLT3-ITD mutation suggested a poor prognosis, although ATRA treatment achieved a good response. MMI administration has since been terminated and thyroid hormone levels remain closely monitored.

To conclude, the current case report presents a patient with hyperthyroidism who was administered PTU for 11 years prior to taking MMI. The patient developed FLT3-ITD-positive APL three months following MMI intake but responded well to ATRA treatment. To the best of our knowledge, this is the first case of a patient with hyperthyroidism concurrent with FLT3-ITD-positive APL. We hypothesize that this case is likely to enrich the understanding of the association between thyroid diseases and hematological disorders.
